# The UFO procedure for prosthetic valve detachment after redo aortic valve replacement in Behcet’s disease: Case report

**DOI:** 10.1016/j.xjtc.2025.01.007

**Published:** 2025-01-19

**Authors:** Chunyan Jiang, Xiangfeng Gong, Hao Niu, Haochen Wang, Wenxuan Shi, Zhenghua Xiao

**Affiliations:** Department of Cardiovascular Surgery, West China Hospital, Sichuan University, Chengdu, Sichuan, PR China

**Figure:**
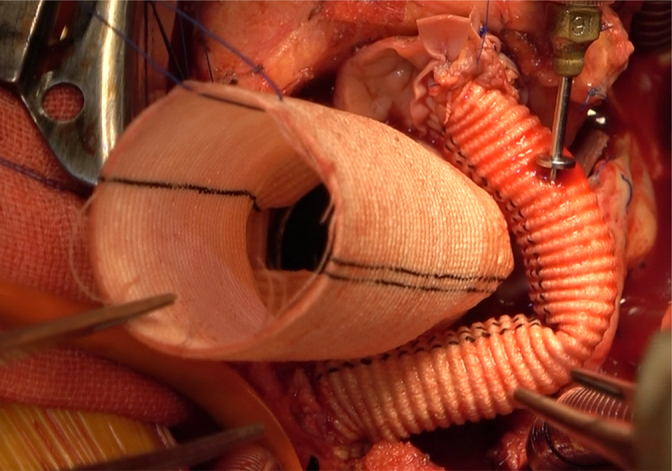
The flanged Bentall plus Cabrol procedure was performed to address complex aortic root lesions.

**Central Message**

UFO surgery provides a relatively radical solution for patients with Behcet disease who experience valve detachment and extensive cardiac damage following redo aortic valve replacement.

Cardiovascular involvement in Behcet disease (BD) occurs in 7% to 46% of cases.^1^ Such complications as valve detachment and pseudoaneurysm frequently follow conventional aortic valve replacement (AVR) owing to tissue fragility and inflammation, leading to high rates of reoperation (78%-100%) and mortality (20%-47.3%).^2^^,^^3^ Valve detachment may recur even after redo AVR, accompanied by extensive damage to the mitral valve and intervalvular fibrous body (IVFB).

Here we report a re-redo case with BD due to aortic detachment, severe mitral regurgitation, and root pseudoaneurysm, treated with the commando, flanged Bentall, and modified Cabrol procedure, collectively referred to as the “UFO” procedure.

## Case Description

A 35-year-old man with a complex past cardiac surgical history was admitted to our department for fatigue. At age 30 years, he had undergone the first AVR combined with mitral valvuloplasty and tricuspid valvuloplasty. During hospitalization, the patient was diagnosed with BD and then started on regular cyclophosphamide and corticosteroid therapy. However, he underwent redo AVR only 4 months later because of aortic prosthesis detachment. Since then, he continued to receive immunosuppressive treatment.

**Figure 1:**
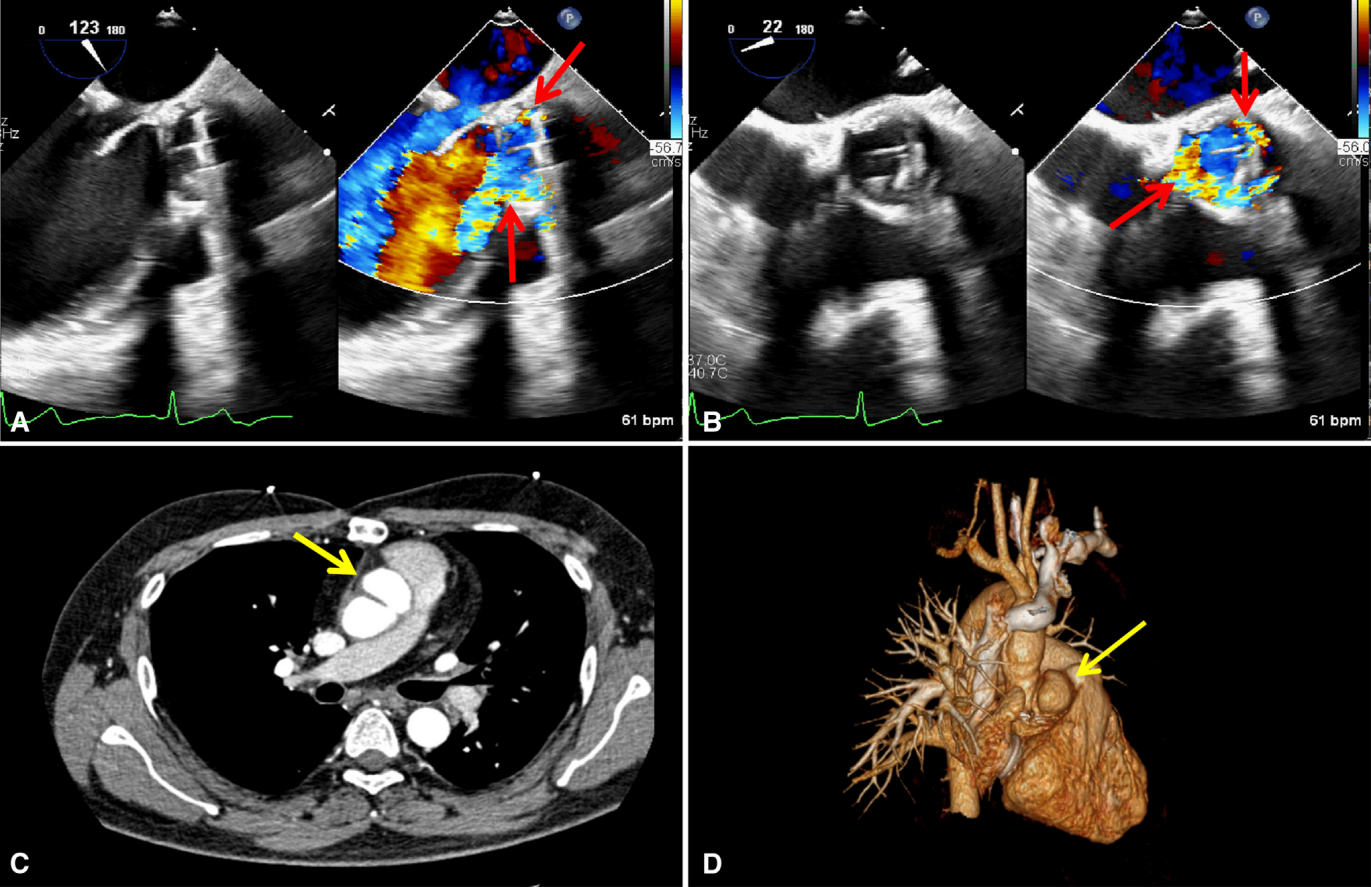
A and B, Preoperative transesophageal echocardiogram demonstrating severe perivalvular regurgitation (*red arrows*). C and D, Computed tomography angiography showing a pseudoaneurysm originating the aortic root (*yellow arrows*).

Transesophageal echocardiography at admission demonstrated severe aortic perivalvular leak and severe mitral regurgitation. Computed tomography angiography revealed a pseudoaneurysm at the aortic root (Figure 1). His C-reactive protein concentration was 50.1 mg/L.

After a thorough discussion of the patient's condition and careful communication with the family, an emergent UFO procedure was planned. Ethical Committee approval was waived, and informed consent for publication was obtained from the patient. Surgery was performed via redo redo-sternotomy. Cardiopulmonary bypass was achieved via ascending aortic perfusion and bicaval drainage. An oblique aortotomy was performed, extending through the noncoronary sinus and the aortic annulus to reach the superior aspect of the left atrium. We observed that the prosthetic aortic valve was detached at the right and left coronary cusps, and there was a pseudoaneurysm in the anterior part of the aortic root communicating with the left ventricle. The diseased ascending aorta and aortic root were excised to the aortic valve annulus level, and the coronary buttons were prepared. Valve detachment led to curtain destruction, with the posterior leaflet of the mitral valve tethered.

**Figure 2:**
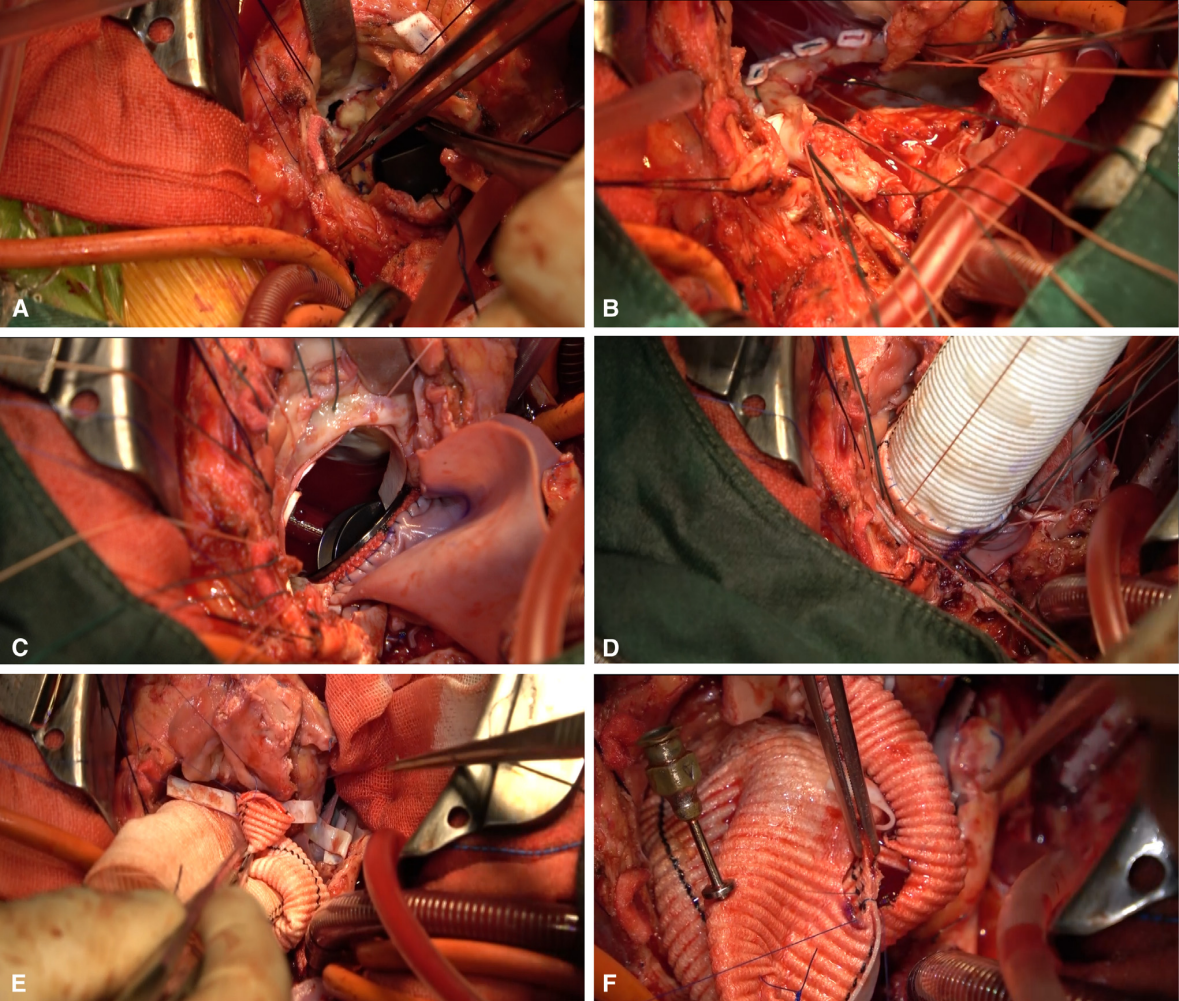
Intraoperative views of the UFO procedure. A, Aortic valve detachment. B and C, Reconstruction of mitral annulus and aortoventricular junction with Commando procedure. D, Implantation of flanged conduit. E and F, Modified Cabrol procedure.

After excision of the pseudoaneurysm, a combined biatrial incision was made from the left atrial roof to the anterior wall of the right atrium. After removal of the mitral annuloplasty ring, the anterior mitral leaflet and subvalvular structures were removed. After carefully sizing the mitral annulus between anterolateral commissure and posteromedial commissure, a 27-mm mechanical prosthetic valve was chosen. The anterior mitral annulus and aortic curtain were reconstructed using a trimmed bovine pericardial patch, while the left atrial roof was reconstructed. Then a 23-mm mechanical valve was inserted into a 28-mm Dacron graft with 1.5 cm of free edge as the “skirt,” forming a flanged valved conduit. Sandwich reinforcement with a bovine pericardial strip and felt pledgets was used to reconstruct the aortoventricular junction before implantation of the flanged conduit. Owing to the dense adhesion of the left and right coronary ostia, the Cabrol technique with an 8-mm graft was used to reimplant the coronary system to the aortic graft (Figure 2, Video 1).

**Figure E1:**
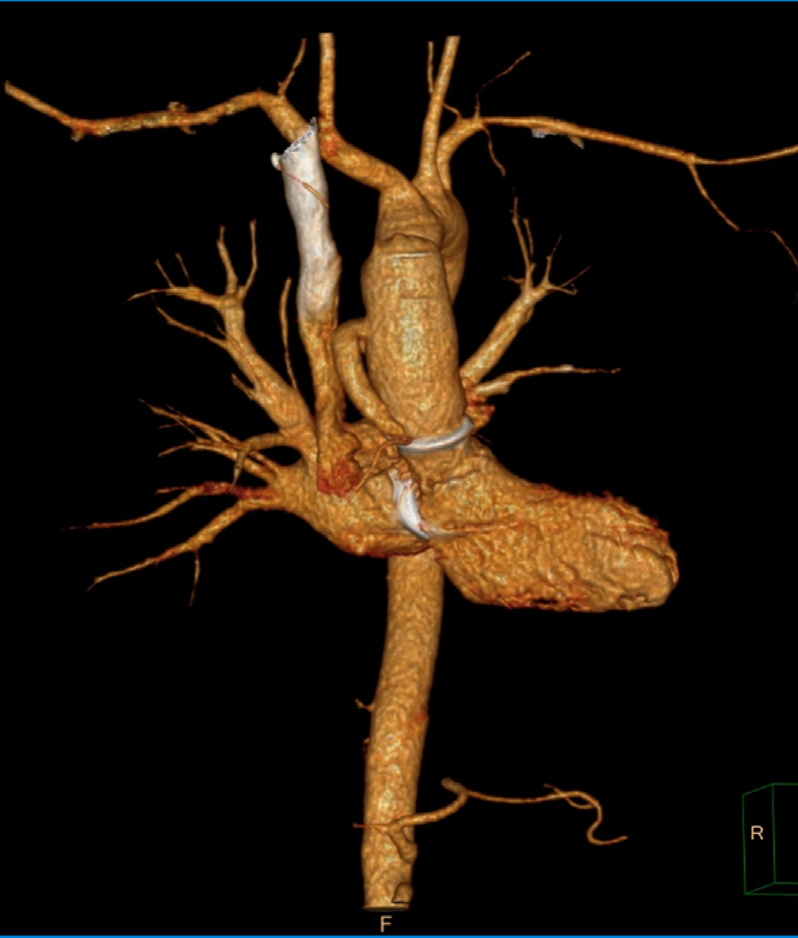
Postoperative computed tomography angiography scan.

Postoperative echocardiography demonstrated satisfactory prosthetic valve mobility. Computed tomography angiography confirmed that the Cabrol conduit was functioning properly, with no contrast agent leakage or pseudoaneurysm (Figure E1). Immunosuppressive therapy was resumed after control of infection. The patient was discharged on postoperative day 14 with no complications. No pseudoaneurysm or valve dysfunction occurred during the 7-month follow-up period.

## Discussion

The appropriate patch size during IVFB reconstruction is critical to prevent left ventricular outflow tract obstruction, which may result from a long curtain that dynamically folds with each heartbeat, causing valve rocking.^4^ The width of the curtain was calculated as the distance between the medial and lateral fibrous trigones, with an additional 8 to 10 mm included to ensure a tension-free patch. Our center recommends a curtain length of 1 to 1.5 cm. Additionally, the flanged Bentall procedure can reduce the tension of the suture ring on the aortic annulus to prevent further prosthetic detachment. More importantly, the flanged conduit also provides a stable aortic prosthesis and part of the neo-IVFB to minimize the risk of valve rocking. Both the sandwich technique and Flanged Bentall procedure were used to prevent anastomotic leakage and pseudoaneurysm formation in patients with BD. The soft Dacron graft with minimal tension on the double- reinforced aortic annulus help keep the annulus structure intact long-term.

In conclusion, the management of BD patients who experience prosthesis dehiscence and extensive cardiac damage following redo AVR remains challenging. Despite its complexity and associated risks, the UFO procedure can provide promising short- and long-term outcomes.

## Conflict of Interest Statement

The authors reported no conflicts of interest.

The *Journal* policy requires editors and reviewers to disclose conflicts of interest and to decline handling or reviewing manuscripts for which they may have a conflict of interest. The editors and reviewers of this article have no conflicts of interest.

## Supplementary Data

Video 1 Preoperative examination and primary steps of the UFO procedure.Video available at: https://www.jtcvs.org/article/S2666-2507(25)00041-0/fulltext.
