# Supplementary figures and images for: The UFO procedure for prosthetic valve detachment after redo aortic valve replacement in Behcet’s disease: Case report
Source: JTCVS Tech. 2025 Jan 19;30:57–61. doi: 10.1016/j.xjtc.2025.01.007 (PMC11998350; doi:10.1016/j.xjtc.2025.01.007)

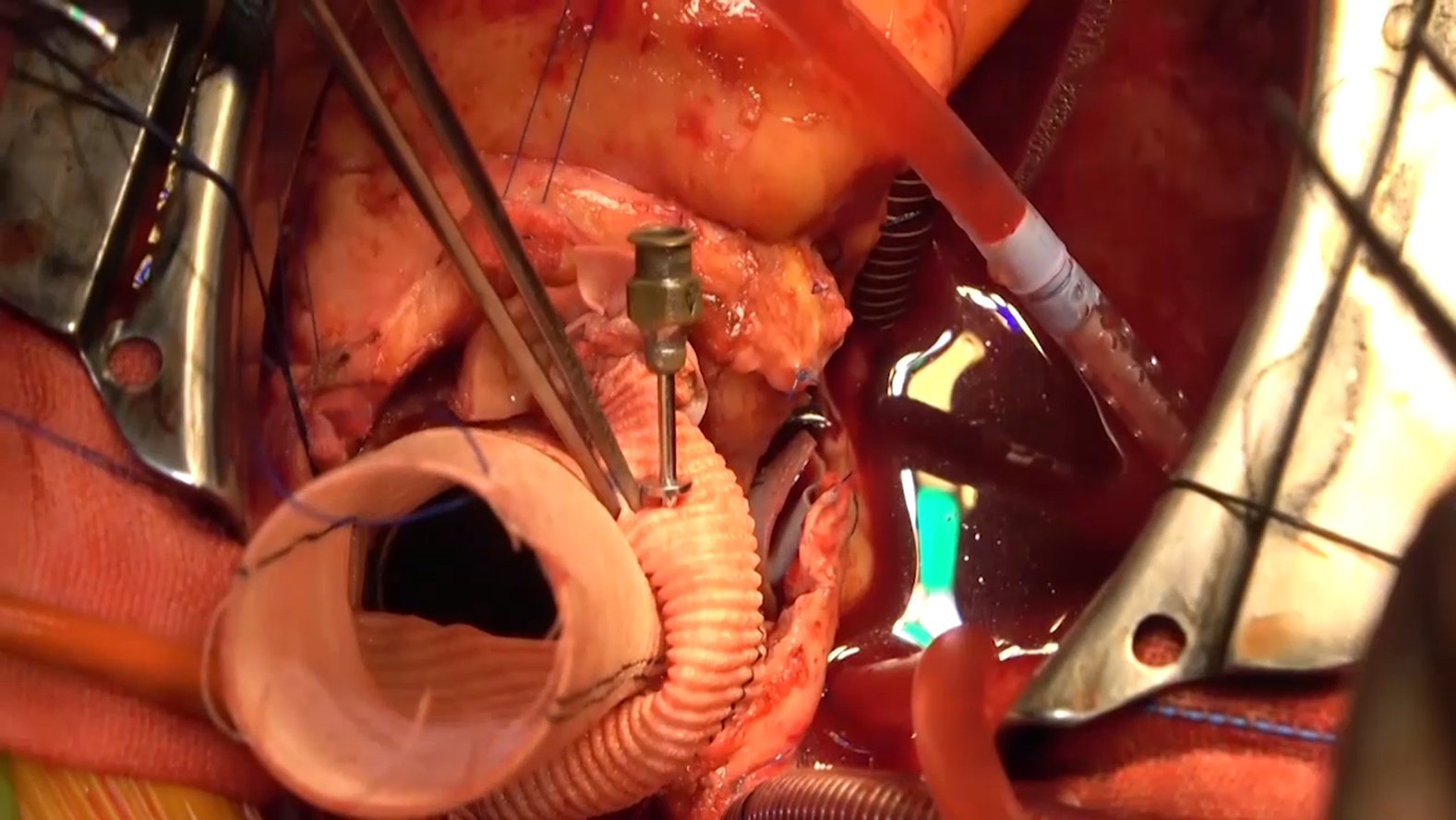

Supplement: Video 1 — Preoperative examination and primary steps of the UFO procedure.Video available at: https://www.jtcvs.org/article/S2666-2507(25)00041-0/fulltext. [file fx2.jpg]
